# Operating table stability and patient safety during an earthquake based on the results of a shaking table experiment

**DOI:** 10.1016/j.bjao.2024.100301

**Published:** 2024-07-15

**Authors:** Takahiko Tsutsumi, Keita Fukuyama, Kazumasa Kishimoto, Yukiko Mori, Osamu Sugiyama, Goshiro Yamamoto, Masahiro Kurata, Ueshima Hiroaki, Kenichi Saito, Tomohiro Kuroda, Shigeru Ohtsuru

**Affiliations:** 1 Graduate School of Medicine and Faculty of Medicine, Kyoto University, Kyoto, Japan; 2 Department of Earthquake Resistant Structures, Disaster Prevention Research Institute, Kyoto University, Kyoto, Japan

**Keywords:** anaesthesiologist, disaster planning, earthquake, operating room, safety management, surgical equipment

## Abstract

**Background:**

The damage that may be caused to the operating table and patients under general anaesthesia when a large earthquake occurs is unclear. We aimed to evaluate the movement and damage to operating tables and patients under general anaesthesia during an earthquake.

**Methods:**

An operating table with a manikin resembling a patient on it was placed on a shaking table, and seismic waves were input into the shaking table. The effects of seismic waves were evaluated by altering surgical positions (supine and head-down positions), operating tables, flooring material, seismic waves, and output. We observed the movement of the operating table and measured the acceleration of the operating table and manikin head.

**Results:**

Under 90% output of long-period seismic waves, the operating table with the supine manikin was overturned. Under experimental conditions that did not cause rocking, shaking such as tilting of the operating table caused stronger acceleration in the manikin's head than in the operating table. There was no clear relationship between operating table rocking and maximum acceleration as a result of programmed seismic waves. In long-period earthquakes, rocking and overturning occurred >60 s after the onset of shaking, whereas in direct earthquakes, rocking occurred within 10 s.

**Conclusions:**

An earthquake could cause strong acceleration of the patient's head under general anaesthesia, and operating tables may overturn or shake violently. Regarding patient safety, further measures to prevent overturning should be considered.

Since 2000, 342 earthquakes of magnitude 7 and greater on the Richter scale, capable of causing tremendous damage, have occurred worldwide.^1^ Large earthquakes have affected many lives and caused enormous economic losses. For example, the Great East Japan Earthquake, during which >18 000 people died, had a magnitude of 9.0.^2^ The August 2016 Central Italy earthquake, which caused the death of 298 people, had a magnitude of 6.2.^3^

Although medical institutions play an important role in treating earthquake-related victims, these institutions can also be seriously damaged by earthquakes.^4^, ^5^, ^6^ Even if the building itself is safe, there may be interruptions in functioning of medical equipment, supplies, and power supply, making regular hospital operations difficult during a major earthquake.^4^^,^^6^

Early earthquakes can also cause significant damage to operating rooms.^6^ At the time of the Great East Japan Earthquake, 474 people were undergoing surgery in 213 hospitals.^6^ Of these hospitals, 154 reported difficulties in performing operations in blackouts and tremors after the earthquake.^6^ From the point of view of safety, patients under general anaesthesia cannot protect themselves in the event of a huge earthquake during their surgery.

The guidelines for medical staff in operating rooms (surgeons, anaesthesiologists, operating room nurses, etc.) state that in the event of an earthquake, the first priority should be to protect themselves and their patients.^7^ Operating tables and anaesthesia machines are cited as items that can be held on to in the event of an earthquake. However, how operating tables and patients on them would shake or be damaged in the event of an actual earthquake has not been verified. Therefore, we considered investigating the safety of operating tables in the event of an earthquake under several conditions necessary.

Studies regarding earthquake damage to medical facilities are mainly of two types: one type evaluates the actual damage to medical facilities caused by earthquakes, and the other type simulates damage to medical facilities and equipment in experimental settings. Previous reports on earthquake damage have included information on damage to operating rooms during earthquakes. However, these reports lacked detailed descriptions about the movement of operating tables.

Shaking table tests have been performed to evaluate earthquake damage to medical equipment, such as medicine cabinets, medicine bottles, and dialysis machines. In these experiments, earthquakes were reproduced using shaking table test equipment; the movement and damage of medical devices during earthquakes were evaluated.^8^, ^9^, ^10^, ^11^ However, to date, no patient operating table has been subject to the shaking table test. In this study, we aimed to evaluate the behaviour of operating tables and ensure patient safety (using a human manikin) during simulated earthquakes through the use of shaking table tests.

## Methods

### Equipment and layout

**Fig. 1:**
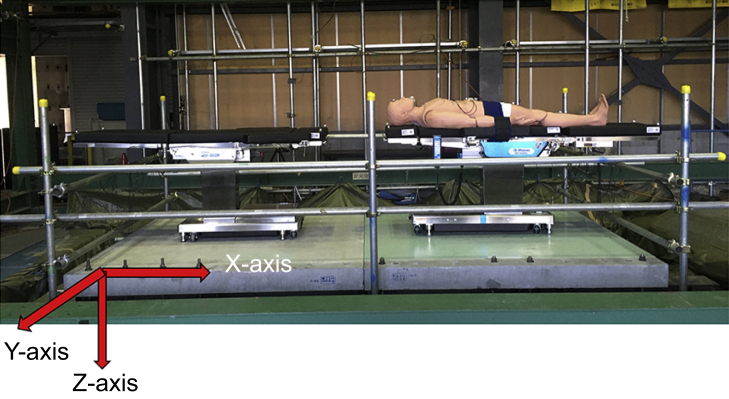
Equipment layout in the experiment. A shaking tabletop is used to reproduce the floor of a building on a device that generates shaking. The operating tables are placed on the tabletop, on which a patient-shaped manikin is placed. The acceleration measurement directions are as follows: the horizontal direction of the screen as the X-axis, the direction from the back of the screen to the front as the Y-axis, and the vertical direction toward the ground as the Z-axis.

Shaking table tests of the operating table and manikin were conducted at the Disaster Prevention Research Institute of Kyoto University, Japan (Fig 1). The shaking table reproduces the shaking movements of an earthquake using programmed seismic waves. The behaviour of the operating table and manikin under seismic motions and the associated acceleration responses were evaluated. The shaking table was 5×5 m in size, had a maximum payload of 300 kN, and provided six degrees of freedom of excitation.^12^ To replicate the floor in a building, a concrete slab was placed on the shaking table, with its right half covered by a multilayer vinyl floor sheet commonly used in operating rooms. Two types of flooring materials, concrete and multilayer vinyl, were used to investigate the effects of floor friction. A safety fence was installed to prevent the operating table from falling off the shaking table.

After setting up the specimen on the shaking table (Fig 1), the programmed seismic waves were generated. The seismic waves applied to the shaking table facilitated the reproduction of the movements of the specimen during an earthquake. For safety reasons, no one was allowed near the shaking table during shaking. The damage and movement of the specimen were observed using attached accelerometers and video recordings.

### Seismic waves

**Fig. 2:**
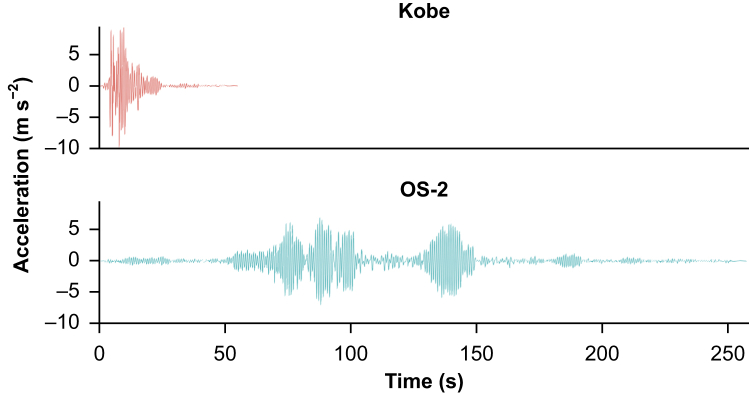
Acceleration of JMA-Kobe and OS-2. JMA-Kobe motion is a seismic motion measured during the 1995 Great Hanshin Earthquake. It consists of abrupt high-intensity pulse-type motion at a period near 1.0 s. The long-period long-duration wave (OS-2) is a programmed motion and has long-period wave components.

Figure 2 shows the seismic waves used in this experiment (i.e. floor acceleration responses) in a low-rise building under earthquake motions. JMA-Kobe motion is a seismic motion measured during the 1995 Great Hanshin Earthquake. The motion consists of abrupt high-intensity pulse-type motion at a period near 1.0 s. The long-period long-duration wave (OS-2) is a synthesised motion used for designing flexible structures assuming a future Nankai Trough earthquake. This wave contains long-period shaking components, such as those observed during the 2011 Great East Japan Earthquake and 1985 Mexico City earthquake, and lasts for a few minutes. Long-period wave components, with periods ranging between 2 and 10 s, take longer to travel back and forth than the pulse-type components in a near-fault seismic motion. For seismic waves, only the acceleration component in the Y-axis direction (short-axis direction of the operating table) in Fig 1 was the input.

We used Japan Metrological Agency (JMA) intensity to represent the scale of the earthquake waves used for this experiment.^13^ The JMA Kobe wave is based on the seismic waves observed during the Great Hanshin Earthquake.^14^ The 100% output of the JMA Kobe wave corresponds to a seismic intensity of 6+, whereas the 70% output corresponds to a seismic intensity of 6−. The OS-2 wave is a long-period seismic wave programmed in anticipation of the Nankai Trough earthquake.^15^ The 100% output of OS-2 corresponds to JMA intensity of 6+, whereas the 70% output equates to JMA intensity of 6−.

At JMA intensity of 6−, it becomes difficult for people to remain standing, and unsecured furniture may topple over. In addition, buildings with low earthquake resistance may tilt. At JMA intensity of 6+, most unsecured furniture is likely to overturn, and buildings with low seismic resistance may collapse.^13^

### Operating tables and manikin

Table 1 presents the details of the two types of operating tables used for the experiment. The operating tables (MOT-5602BW and MOT-VS600Dj, Mizuho Company, Tokyo, Japan; hereafter referred to as OT-A and OT-B, respectively) used for these tests were moveable but differed in size and braking performance. The brake mechanisms in both tables include a fixation with four brake legs. A hydraulic damper was attached to OT-B but not to OT-A (Table 1). These operating tables are commonly used in Japan and are also used in North America and Asian countries. When the operating table is fixed, only the bottom of the brake is in contact with the floor. In this experiment, we used one manikin as a simulated patient. The manikin (W44619; Human Body, Ibaraki, Japan) was 177 cm tall and weighed 75 kg (Table 1), replicating the height and weight of a standard male adult. In the supine position, the manikin's abdomen and upper limbs were secured using cloth belts. In the head-low position, the manikin was secured using shoulder pads and a clothing belt.

**Table 1:** Details of the operating table and manikin.

Model number of the operation table	MOT-5602BW	MOT-VS600Dj
Name in the study	OT-A	OT-B
Weight (kg)	323	390
Measurement of the tabletop (mm)	1950×500	2124×500
Measurement of the table base (mm)	973×483	1133×483
Range of height (mm)	520–1000	520–1000
Fixation mechanism	With four brake legs	With four brake legs
Hydraulic damper	Without	With
Supplier	Mizuho Company, Tokyo, Japan	

Model number	W44619	
Weight of the manikin (kg)	75	
Height of the manikin (cm)	177	
Supplier	HUMANBODY.JP	
Link	https://humanbody.jp/guide/ctrg/hblg-resucue_v09set.pdf	

### Experimental conditions

Table 2 presents the conditions tested in these experiments, which comprised five elements: type of operating table, surgical position, type of flooring, type of seismic wave, and output of the seismic wave.

**Table 2:** Experiment condition.

	Operating table	Surgical position	Height of operating table (mm)	Floor material
Experiment 1	OT-A	Supine	650	Concrete
Experiment 2	OT-A	Head-down	650	Concrete
Experiment 3	OT-B	Head-down	650	Concrete
Experiment 4	OT-A	Head-down	650	Multilayer vinyl
Experiment 5	OT-B	Supine	650	Concrete

The operating tables OT-A and OT-B have been described in the previous section. The height of the table was set at 650 mm in both positions. The two tested positions were the supine position used in general open surgery and head-down position used in urological and gynaecological laparoscopic surgeries. The tabletop was kept in a horizontal position when the test manikin was placed supine and with a 25° downward tilt when the test manikin was placed in the head-down position. Two types of flooring were used: a concrete floor and a floor covered with multilayer vinyl floor sheet.

In Experiments 1–5, we input three or four programmed seismic waves. In Experiment 1, JMA-Kobe 70%, OS-2 70%, OS-2 80%, and OS-2 90% were generated once. In Experiments 2–5, JMA-Kobe 70%, OS-2 70%, and OS-2 80% were generated once each.

### Measurements

**Fig. 3:**
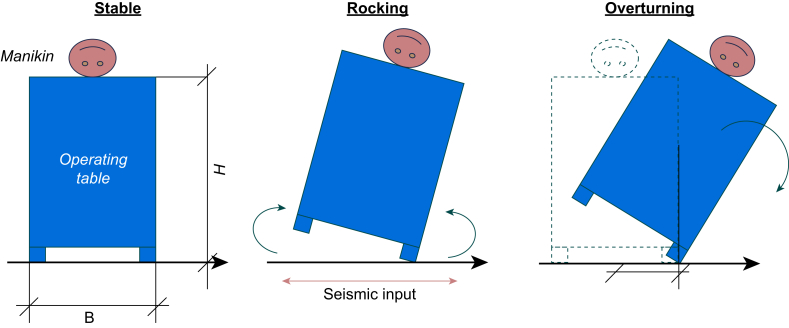
Seismic movement of the operating table. This illustration shows the three movement patterns of the operating table during an earthquake: stable, rocking, and overturning. B refers to the length of the short side of the operating table base; H refers to the height from the floor to the top of the operating table.

In this experiment, we measured acceleration and observed the dynamics of the operating table to verify the damage to the operating table and patients under general anaesthesia during an earthquake. Accelerometers were installed on the forehead of the manikin, side of the operating table, and shaking table, and changes in acceleration were observed over time. We observed whether the operating table rocked, overturned, or showed tremors during the period from the start to the end of the shaking. The definitions of rocking and overturning were based on a study by Cosenza and colleagues^16^ The movement of the legs of the operating table leaving the ground was considered rocking, and the motion of the table falling sideways was called overturning (Fig 3). In this study, we defined shaking of the operating table without movement of the operating table feet as tremor. The shaking stopped when the operating table fell. In this experiment, the point at which rocking started was defined as the point at which the acceleration in the z-axis direction of the accelerometer exceeded 2 m s^−2^.

### Analysis environment

Analysis and visualisation were performed in a pre-built environment using R (version 4.3.0),^17^ Python (version 3.9.7),^18^ and Jupyter Lab (version 3.6.3)^19^ on Windows 10 Home. The code for each stage of extraction and aggregation was reposited on GitHub (https://github.com/medinfo2/earthquake_st).

## Results

### Results by experimental condition

Table 3 summarises the results of the 16 patterns of the shaking table experiments, acceleration of the operating table and manikin head, and movement of the operating table observed in the video. Of the 16 patterns, rocking occurred in eight conditions, either in isolation (*n*=6) or in combination with overturning (*n*=1) or touching the safety bar (*n*=1). Tremor (no rocking) occurred in eight conditions, either in isolation (*n*=5) or in combination with touching the safety bar (*n*=3).

**Table 3:** Experiment results.

Experiment No.	Condition			Seismic wave		Result				
	Operating table	Surgical position	Floor material	Type	Output (%)	Table movement	Time to rocking (s)	Maximum acceleration		
								Shaking table (m s^−2^)	Operating table (m s^−2^)	Forehead of manikin (m s^−2^)
1	OT-A	Supine	Concrete	Kobe	70	Rocking	4.6	6.5	71.9	43.8
				OS-2	70	Rocking	87.4	4.6	53.5	28.7
					80	Rocking	87.2	5.1	75.2	36.6
					90	Rocking/overturn	73.9	4.9	NA	NA
2	OT-A	Head-down	Concrete	Kobe	70	Rocking	4.2	6.4	64.5	29.5
				OS-2	70	Tremor	NA	5.1	8.1	11.4
					80	Rocking	75.6	5.2	74.8	37.8
3	OT-B	Head-down	Concrete	Kobe	70	Tremor	NA	7	8.2	11.8
				OS-2	70	Tremor	NA	5.1	6.1	8.3
					80	Tremor/safety bar	NA	NA	6.6	10.9
4	OT-A	Head-down	Multilayer vinyl	Kobe	70	Tremor	NA	7.1	8.1	33.4
				OS-2	70	Tremor/safety bar	NA	NA	5.7	16
					80	Tremor/safety bar	NA	NA	7.2	16.1
5	OT-B	Supine	Concrete	Kobe	70	Rocking	9.4	7.2	39.8	22.3
				OS-2	70	Tremor	NA	5.1	6.6	10.3
					80	Rocking/safety bar	74.0	NA	68.6	28.7

### Overturned table

**Fig. 4:**
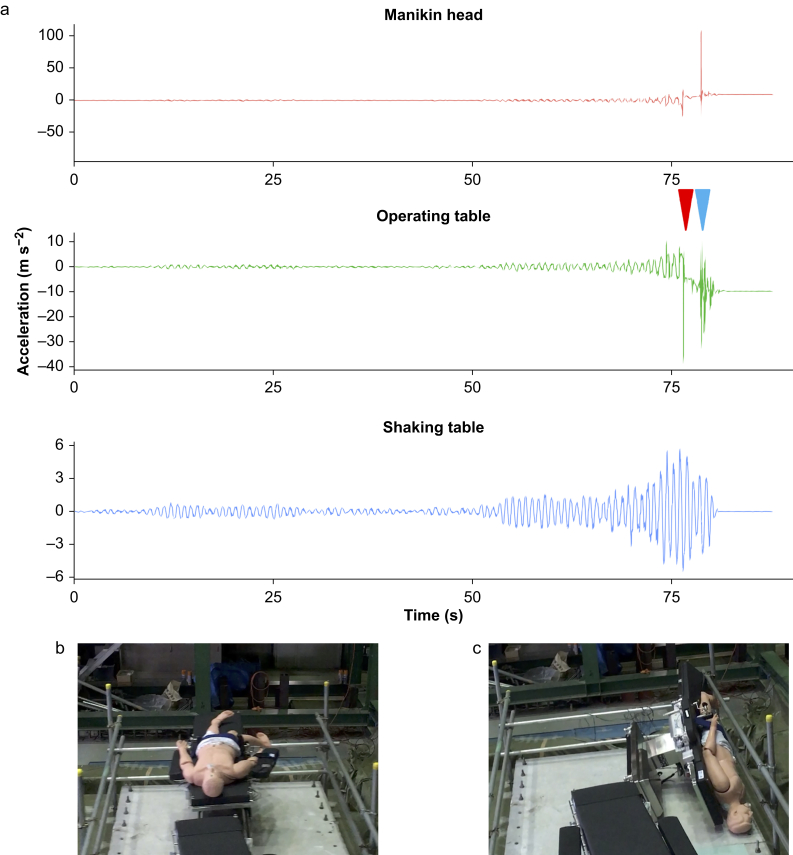
Outline of an experiment where the operating table overturned. (a) Timeline of acceleration. The red triangle indicates the point at which the rocking began. The blue triangle indicates the point of overturning. (b) Image of the start of rocking. (c) Image of the overturning operating table. (For interpretation of the references to color in this figure legend, the reader is referred to the Web version of this article.)

Figure 4 shows an outline of the experiment in which the operating table was overturned. In the experiment, a long-period vibration of 90% was applied to OT-A in the supine position with a concrete floor. The operating table, manikin, and shaking table shook synchronously until ∼75 s after the shaking started. At the red triangular point in Figure 4, the acceleration of the operating table suddenly increased, and the video confirmed that rocking began from this point. The operating table was overturned after the rocking continued. The last erratic change in the operating table acceleration was as a result of the operating table hitting the safety bar or ground during the fall.

### Acceleration by operating table rocking status

**Fig. 5:**
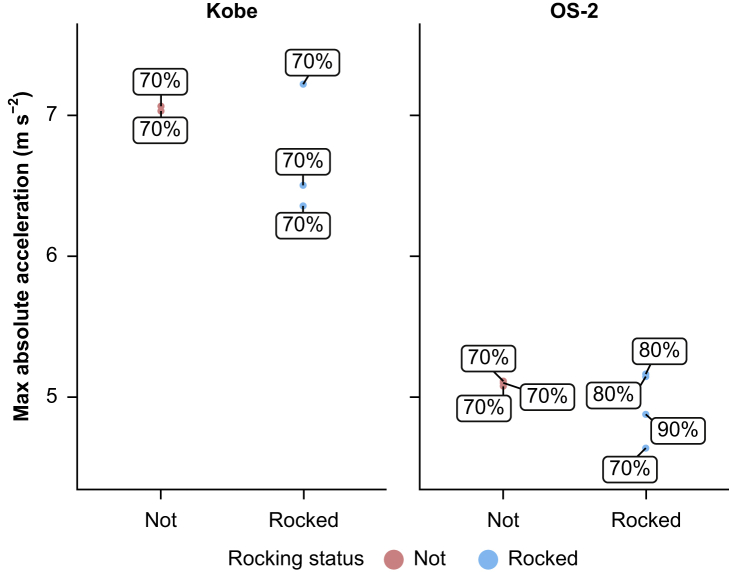
Relationship between maximum measured acceleration and presence or absence of rocking. The figure shows the maximum applied acceleration and presence or absence of rocking under the two seismic waves. Labels indicate the output intensities of each seismic wave.

Figure 5 shows the results of the 12 patterns of verifying the maximum measured acceleration and presence or absence of rocking, excluding the four patterns in which the fence did not trap the table. In the rocking example, the shaking table acceleration was evaluated until just before the rocking started. In the non-rocking example, the shaking table acceleration was evaluated until the end of shaking. No clear relationship between maximum acceleration and rocking was observed for Kobe or OS-2 in this study.

### Comparison of acceleration of the operating table to that of the manikin head

**Fig. 6:**
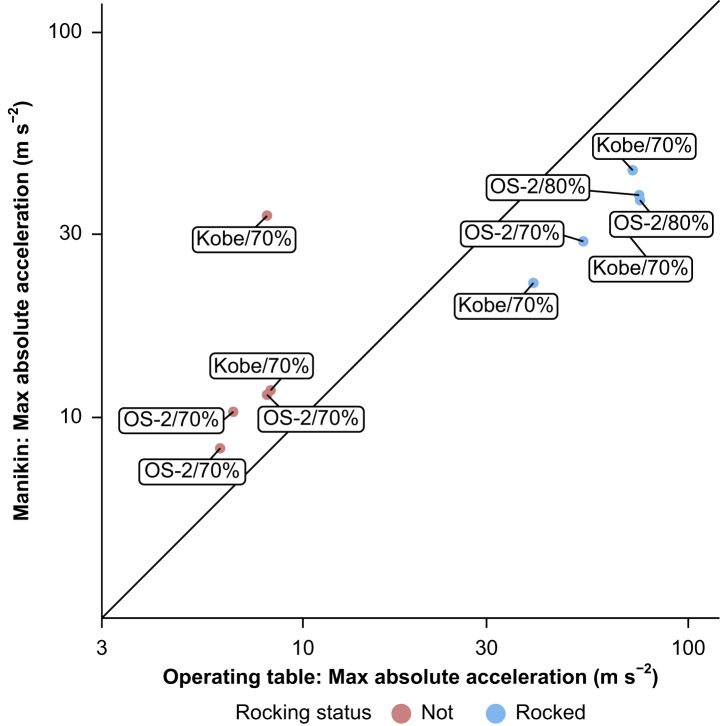
Maximum acceleration in the operating table and manikin head. The figure shows the maximum measured acceleration in the operating table and manikin head. The labels indicate the output intensities of each seismic wave. M, manikin head; OT, operating table. The diagonal line indicates OT=M. Both the vertical and horizontal directions of the graph are logarithmic.

Except for the experiment in which the operating table was overturned, the relationship between the maximum acceleration measured from the operating table and manikin head for 15 patterns in which the operating table was not overturned is shown in Figure 6. In this evaluation, the changes in acceleration during rocking were also included in the calculation. Under experimental conditions that did not cause rocking, a stronger acceleration was observed in the manikin head than in the operating table itself.

## Discussion

The prospect of a massive earthquake during surgery under general anaesthesia is rare. Therefore, conducting high-quality observational studies is not easy. How operating tables and patients would shake or the extent of acceleration of the patient's head while lying on the operating table during an earthquake remain unclear. If the operating table were to shake or fall, care providers would be challenged to protect patients under general anaesthesia from harm. To identify measures to protect patients, investigating how the operating table would move and be accelerated during an earthquake is important. In this study, we found that both long-period earthquakes and direct earthquakes can cause rocking or turnover of the operating tables. The heads of the patients would be exposed to strong acceleration at an intensity that could realistically occur. These results suggest various risks in operating rooms during earthquakes, including not only patient falls, but also accidental extubation and trauma to patients and medical staff by surgical instruments.

Table 3 lists the absence of a clear relationship between rocking or falling and maximum acceleration. In this study, we examined floor materials, body position, and type of operating table as factors other than maximum acceleration. However, we could not establish whether these factors contributed to overturning or rocking. This may be attributed to the limitation that only 16 experimental studies could be conducted. Moreover, in four of these experiments, contact with the safety fence prevented the results from being used for verification. However, conducting additional shaking table experiments was difficult because of time and financial constraints.

The factors contributing to operating table rocking are complex. For a cuboid rigid body, the rocking limit acceleration depends on the height of the centre of gravity and length of the scaffold from just below the centre of gravity.^20^ However, in this experiment, there was no obvious effect of operating tables differences and maximum acceleration on the occurrence of rocking. Operating tables with a manikin are not cuboid rigid bodies. As preliminary experiments, we conducted shaking table tests without a manikin in experiment numbers 2, 3, 4, and Kobe 70% of 5. No occurrence of rocking was observed in these cases. The patient's mass, 75 kg in these experiments, is thought to have an important effect on the occurrence of operating table rocking. To elucidate the conditions under which operating tables are prone to shaking during earthquakes, we examined factors such as seismic intensity, types of operating tables, floor materials, and surgical position. However, none of these factors showed a clear influence on susceptibility to shaking. Additionally, during real surgical settings, various instruments such as drapes, i.v. poles, instrument tables, and medical staff are present around the operating table and patient. The impact of these factors on the occurrence of rocking or overturning of operating tables remains unknown. Further research is warranted to determine the conditions required to minimise the likelihood of operating tables being rocked during an earthquake.

As presented in Table 3, direct earthquake causes rocking within 10 s from the start of shaking in the rocking group. In contrast, long-period ground motions caused rocking after >60 s. Larger earthquakes cause longer duration of shaking. Even after medical staff sense the shaking, determining the scale and type of the earthquake is difficult. These results suggest that if staff safety can be assured, it is desirable for staff to stabilise the operating table within seconds of sensing shaking. The operating table should be stabilised to protect patients until the earthquake subsides.

The efficacy of preventive measures could not be validated, owing to the limited number of experiments conducted. However, to prevent observed instances of rocking or overturning, it is suggested that (1) medical staff around the operating table should stabilise the operating table, (2) the operating table should be secured to the floor with fixtures, and (3) implantable operating tables should be utilised. However, considering that occurrence of earthquakes during surgery is extremely rare, implementing expensive preventive measures solely for this purpose is impractical. Based on variations in hospital seismic structures and earthquake frequency across medical facilities, each institution may assess preventive measures based on the findings of this study.

When medical staff hold down an operating table, holding it from the shorter sides rather than the longer ones might be better to prevent the staff from being trapped under the table or their feet being caught in its base. Furthermore, considering that rocking occurred after >60 s during long-period earthquakes, holding down the operating table until shaking has completely stopped is advisable, even if tremors are minor. However, in this experiment, we were not able to verify the method for medical staff to hold down an operating table safely. Further studies are needed to determine more secure measures.

Under experimental conditions that did not cause rocking, a stronger acceleration was observed in the manikin head than in the operating table. As summarised in Table 3, even in the non-rocking group, the manikin's head was subjected to an acceleration of 8.3–33.4 m s^−2^, which is equivalent to 0.84–3.41 times the standard gravitational acceleration (9.81 m s^−2^). If such accelerations were to occur in an intubated patient connected to a ventilator under general anaesthesia, accidental extubation or disconnection of the intubation tube from the anaesthesia machine may happen. In this experiment, only the operating table and the manikin were included; reproducing the movement of the anaesthesia machine and a manikin was impossible. However, in previous earthquake disaster reports, unfixed medical equipment in the operating room moved significantly during an earthquake.^21^ Furthermore, in the survey of anaesthesiologists after the Sichuan earthquake, 81% immediately replaced the ventilation machine with a bag valve mask.^22^

### Limitations

We could apply the shaking test only once for each experimental condition. Although conducting repeated experiments or retests is desirable for result reproducibility, we faced difficulty in performing them for the following three reasons. First, if the specimen is damaged as a result of overturning, continuing the experiment becomes difficult. Because of the nature of the experiment, if the specimen overturns and is damaged, its structure and properties change, making continuing the experiment difficult. Operating tables are very expensive; consequently, preparing multiple specimens was difficult. Second, previous studies that mention the damage to operating tables during earthquakes are lacking, making determining under what conditions the operating table would experience overturning or rocking difficult. Therefore, we had to verify various conditions within a limited number of experiments. Third, shaking table tests are expensive and require a long preparation period. Owing to these factors, conducting repeated experiments or retests was difficult. However, through this experiment, we have demonstrated the previously unknown unverified fact that operating tables may experience rocking or overturning during earthquakes.

### Conclusions

Our research has brought to light the potential danger of operating tables overturning or rocking during earthquakes, posing a significant risk to patient safety in operating rooms. These findings underscore the importance of conducting additional research into earthquake preparedness in healthcare facilities, with a focus on developing effective strategies for minimising the hazards associated with the movement of medical equipment. By taking proactive steps to address these concerns, we could enhance the safety of both patients and medical professionals during earthquakes, while also bolstering the overall resilience of healthcare organisations operating in earthquake-prone regions.

## Authors’ contributions

Conception of the study: TT, FK, KM.

Study design: TT, KM.

Data collection: TT, KM.

Data analysis: TT, KK, SO, FK.

Drafting the manuscript: TT MY, FK.

Editing and approval of the manuscript: TT, FK, KK, MY, YG, SK, UH, KT, SO.

## Funding

This work was supported in part by the Tokyo Metropolitan Resilience Project of the National Research Institute for Earth Science and Disaster Resilience (NIED) (Subject C-3 leader: Masahiro Kurata).

## Declaration of interest

The authors declare that they have no conflicts of interest.
